# Segmentation of mature human oocytes provides interpretable and improved blastocyst outcome predictions by a machine learning model

**DOI:** 10.1038/s41598-024-60901-1

**Published:** 2024-05-08

**Authors:** Jullin Fjeldstad, Weikai Qi, Nadia Siddique, Natalie Mercuri, Dan Nayot, Alex Krivoi

**Affiliations:** 1 Clinical Embryology and Scientific Operations, Future Fertility, 3 Church St, Toronto, ON M5E 1A9 Canada; 2 Data Science, Future Fertility, 3 Church St, Toronto, ON M5E 1A9 Canada; 3 Chief Medical Officer, Future Fertility, 3 Church St, Toronto, ON M5E 1A9 Canada

**Keywords:** Developmental biology, Embryology, Computational biology and bioinformatics, Machine learning

## Abstract

Within the medical field of human assisted reproductive technology, a method for interpretable, non-invasive, and objective oocyte evaluation is lacking. To address this clinical gap, a workflow utilizing machine learning techniques has been developed involving automatic multi-class segmentation of two-dimensional images, morphometric analysis, and prediction of developmental outcomes of mature denuded oocytes based on feature extraction and clinical variables. Two separate models have been developed for this purpose—a model to perform multiclass segmentation, and a classifier model to classify oocytes as likely or unlikely to develop into a blastocyst (Day 5–7 embryo). The segmentation model is highly accurate at segmenting the oocyte, ensuring high-quality segmented images (masks) are utilized as inputs for the classifier model (mask model). The mask model displayed an area under the curve (AUC) of 0.63, a sensitivity of 0.51, and a specificity of 0.66 on the test set. The AUC underwent a reduction to 0.57 when features extracted from the ooplasm were removed, suggesting the ooplasm holds the information most pertinent to oocyte developmental competence. The mask model was further compared to a deep learning model, which also utilized the segmented images as inputs. The performance of both models combined in an ensemble model was evaluated, showing an improvement (AUC 0.67) compared to either model alone. The results of this study indicate that direct assessments of the oocyte are warranted, providing the first objective insights into key features for developmental competence, a step above the current standard of care—solely utilizing oocyte age as a proxy for quality.

## Introduction

Infertility affects millions of individuals globally (one in six people), necessitating further efforts to improve evaluations and treatments^1,2^. The demand for in vitro fertilization (IVF) as a treatment for infertility is high with over 2.5 million treatments performed annually, however, it has a low success rate (≤ 30% per cycle)^3,4^. A typical IVF treatment comprises exogenous hormonal stimulation to elicit the maturation of several oocytes in the patients’ ovaries, followed by the surgical retrieval of the oocytes through ultrasound-guided intravaginal ovum pick-up and the generation in vitro of embryos by joining sperm and oocytes in the clinical embryology laboratory. A successful IVF treatment comprises of the formation of a viable embryo capable of implanting and further developing in the uterus, which is dependent on the quality of both sperm and oocytes. Following fertilization in vitro, the genetic material and a small amount of its associated RNAs and proteins are all that remains of the sperm, whereas the oocyte contributes its genetic material, cytoplasmic organelles, cell membrane, and a store of maternal mRNA, proteins and metabolic substrates that support early embryonic development^5^. Embryos are typically cultured in nutrient media within the laboratory until development into a blastocyst stage embryo (5–7 days post-fertilization)—weeding out lower quality embryos at earlier stages, with limited potential to implant^6,7^. Given the importance of the oocyte, there are many clinical contexts in which knowing the quality of oocytes in advance of blastocyst development is of critical importance—most notably, in cases of oocyte cryopreservation, donation, or selection (for certain jurisdictions). Understanding oocyte quality can additionally help assess the limiting factors in an IVF treatment and may elucidate why an IVF treatment failed.

Oocytes are a precious biological specimen and have proven difficult to investigate. Previous attempts to establish visual grading schemes to non-invasively evaluate oocyte quality have been unsuccessful due to the poor prognostic value of visible morphological (structural) abnormalities^8^. Moreover, it is not possible to visually assess whether the oocyte has completed its cytoplasmic maturation program, which is to occur in coordination with nuclear maturation—the capacity to resume meiosis and develop into a metaphase II (MII) oocyte^9^. Synchrony between cytoplasmic and nuclear maturation is thought to often be disrupted in oocytes collected from controlled ovarian stimulation cycles, thus a MII oocyte may have delayed cytoplasmic maturation, which could impair the molecular events needed to develop into a good quality embryo^10^. Embryologists ascertain nuclear maturation achievement during the preparation of oocytes for intracytoplasmic sperm injection (ICSI) or cryopreservation; wherein the removal of surrounding somatic cells (cumulus cells) facilitates an unobstructed view of the oocyte’s constituent elements: ooplasm, perivitelline space (PVS), zona pellucida (ZP), and, when mature, an extruded first polar body (PB). Despite this clarity, limited insights have been garnered regarding the specific contributions of these components to developmental competence. However, capturing images of denuded oocytes is ideal for utilization in developing an artificial intelligence (AI) model to evaluate oocyte quality. Furthermore, with the absence of a current standard of care to assess oocyte quality and the crucial role of the oocyte in embryo development, there is a unique case in exploring how AI can address gaps in fertility care and knowledge.

We recently described the development and validation of a deep learning (DL) model that evaluates oocyte images to generate a prediction of blastocyst development. Our model achieved an area-under-the-curve (AUC) of 0.64 and displayed robust performance across diverse IVF demographics, representing an important first step in solving the problem of oocyte classification^11^. However, DL models learn from raw data, which removes the need for feature engineering—an advantage for tasks where there are significant knowledge gaps in terms of which features are relevant, but also a disadvantage, as it results in a lack of interpretability and transparency. An interpretable AI model would not only promote greater trust in its predictions, but also inform future research directions. Thus, the field would benefit from an interpretable oocyte assessment model, aiding both clinical care and research applications.

Previous investigations have assessed the correlation of oocyte morphometry parameters to patient factors such as maternal age (currently used as a proxy for oocyte quality) or to downstream laboratory outcomes, such as successful fertilization and appropriate embryo development—providing some evidence to support the use of quantifiable features in assessing oocyte quality. However, very few features were investigated in these studies, presenting an opportunity for further work. Basic geometric features (e.g., perimeter, area), shape descriptors (e.g., circularity, roundness), and measures of curvature (e.g., solidity) are examples of objective morphometric parameters often assessed in two-dimensional cell shape analysis and can prove informative of cellular function^12^. Small sample sizes and single-center design in these prior studies unfortunately limited the generalizability of any insights gained to other patient populations. Automating the process of measuring oocyte features using AI has the potential to improve accuracy, which is crucial in effectively assessing how downstream reproductive outcomes relate to these measurements. Additionally, correlating reproductive outcomes from a large number of patients across multiple centers will enrich the field’s understanding of oocyte quality and ensure generalizability to multiple clinical settings and populations.

However, there are several challenges in developing an image-based AI model for oocyte assessment. More generally, healthcare datasets are sensitive and under strict regulations, and therefore, difficult to obtain. Within the IVF field specifically, many clinics still collect data in paper form, and the data amalgamated at the global and national level is rarely suitable for research purposes^13^. Addressing specific clinical problems in IVF with AI is complicated by the paucity of publicly available datasets. Only recently did an image dataset of human blastocysts annotated with clinical outcomes become publicly available^14^. Publicly available datasets of oocytes with segmentation ground truths exist, but there are none with clinical annotations—necessitating an extensive data collection process to develop the AI model described in this study^15^.

In the present work, we propose a workflow in which images of denuded MII oocytes from intracytoplasmic sperm injection (ICSI) cycles undergo automatic segmentation via a convolutional neural network (CNN) into its principal regions (ooplasm, ZP, and PVS) and subsequent morphometric feature extraction. The segmented regions of the oocyte will be referred to as masks. A combination of features extracted from the masks and patient variables were used to predict the probability of an oocyte developing into a blastocyst-stage embryo. Both traditional machine learning (ML) and DL models utilizing the masks as inputs were developed and compared, with performance of an ensemble model evaluated to assess whether oocyte evaluation benefits from both approaches.

## Results

### Performance of segmentation model

**Figure 1:**
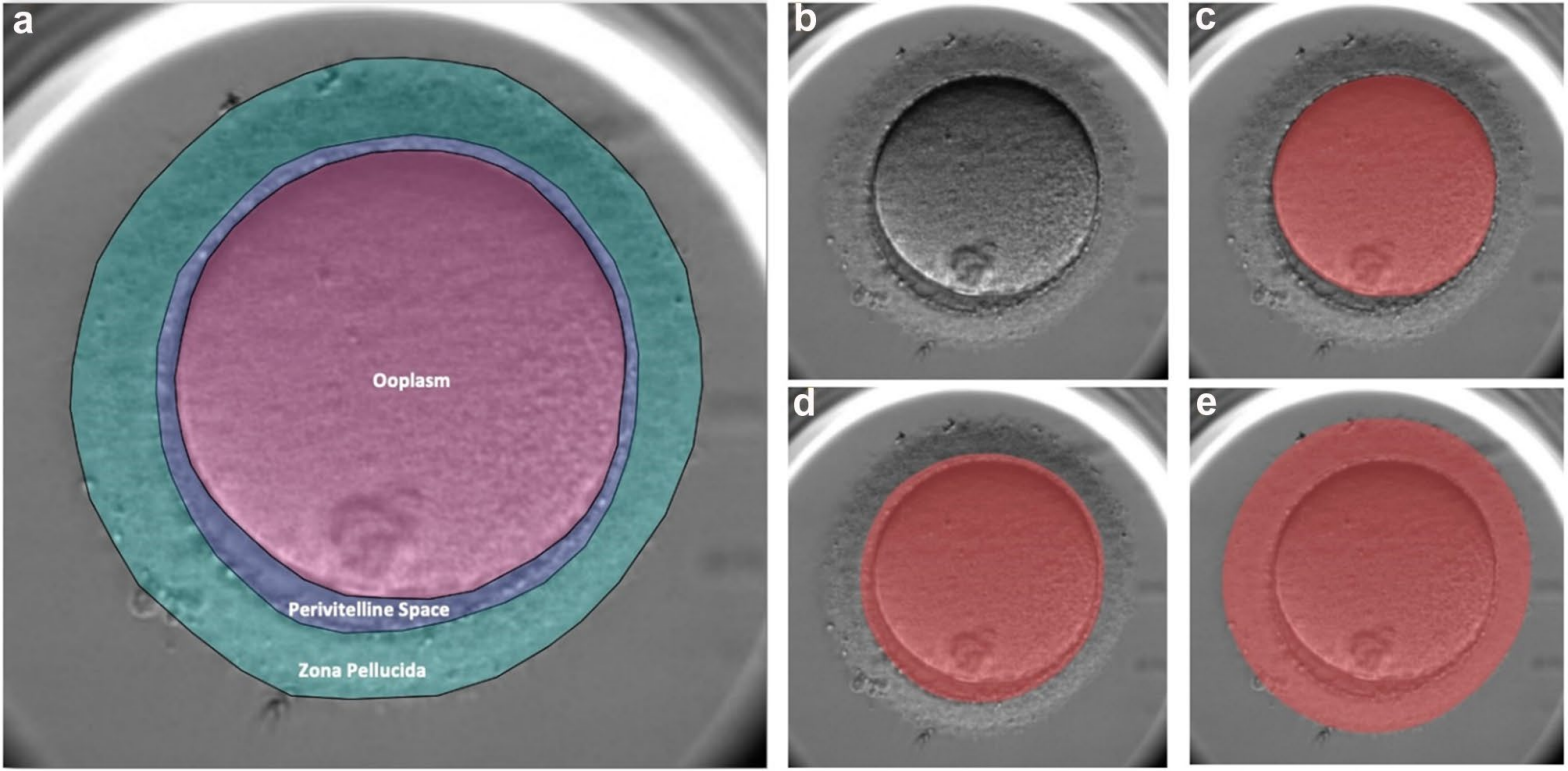
Segmentation of the ooplasm, perivitelline space, and zona pellucida. (**a**) Ground truth labels assigned by embryologists. (**b**) Unsegmented oocyte. (**c**) Segmentation of ooplasm. (**d**) Segmentation of perivitelline space. (**e**) Segmentation of zona pellucida.

Accurate segmentation is critical for our workflow, as the downstream steps to predict blastocyst development are dependent on the quality of the oocyte masks. For the FCBFormer segmentation model, the average IoU scores were 98.1 ± 0.1%, 97.4 ± 0.6%, and 97.0 ± 0.8% for the ooplasm, PVS, and ZP respectively—indicating a high percentage of overlap between ground-truth labels and model-generated masks. These IoU scores also demonstrate an improvement over the previous multi-class segmentation U-Net model, where the IoU scores were 97.8 ± 0.1%, 96.2 ± 0.7%, and 94.7 ± 0.9% for the ooplasm, PVS, and ZP respectively. Examples of embryologist-assigned labels and masks generated by the model are visualized in Fig. 1.

### Feature analysis

Oocytes that developed into blastocysts had a significantly lower mean age (34.9 ± 4.5 years vs. 35.5 ± 4.5 years; P < 0.001) and generally originated from larger cohorts (mean 9.7 ± 5.0 oocytes vs. mean 9.0 ± 5.1 oocytes; P < 0.001). Formulas for calculated features are described in Table 1. Features were compared between oocytes that developed into blastocysts and those that failed to develop, the top ten of which are summarized in Table 2 (information on all features are available in Supplementary Table S1). Nearly all features display significant differences in mean between blastocyst-positive and blastocyst-negative oocytes, with cohort averages for ZP vs PVS relative features as the exceptions. A trend consistently observed across features is that the range of values is broader for blastocyst-negative oocytes, possibly indicating a narrower range of acceptable values for developmentally competent oocytes. However, this trend may be explained by an imbalanced dataset (40.4% blastocyst-positive, 59.6% blastocyst-negative).

**Table 1:** Overview of measurements calculated for ooplasm, ZP, and PVS masks.

Measurement	Description	Formula
Aspect ratio	Ratio of major to minor axis of enclosing best-fit ellipse; higher aspect ratio is indicative of more elongated morphology	${R}_{A}= \frac{{L}_{maj}}{{L}_{min}}$
Circularity	Relation between area and perimeter of mask; maximum value of 1 if object is a perfect circle, < 1 indicative of more oblong shape	$C= \frac{4\pi A}{{L}_{p}^{2}}$
Roundness	Relation between area of mask and area of enclosing ellipse; similar to circularity but accounts for irregular boundaries	$R= \frac{4A}{\pi {L}_{maj}^{2}}$
Solidity	Area of mask divided by area enclosed by convex hull-describes the degree of convexity or concavity of a shape, with greater deviations from 1 representing greater concavity	$S= \frac{A}{{A}_{hull}}$

**Table 2:** Top 10 morphometric measurements for oocytes that did and did not develop into blastocysts in model development.

Feature	Mean ± sd (range) for blastocyst positive samples	Mean ± sd (range) for blastocyst negative samples	p-value
Ooplasm vs PVS major axis ratio	0.892 ± 0.039 (0.658–0.981)	0.899 ± 0.045 (0.599–0.998)	P < 0.001
Ooplasm vs PVS perimeter ratio	0.897 ± 0.031 (0.679–0.993)	0.902 ± 0.037 (0.629–1.011)	P < 0.001
Ooplasm solidity	0.993 ± 0.002 (0.942–0.996)	0.993 ± 0.003 (0.85–0.996)	P < 0.001
Ooplasm roundness	0.963 ± 0.031 (0.484–0.999)	0.958 ± 0.037 (0.469–1.000)	P < 0.001
Ooplasm vs ZP major axis ratio–relative cohort	-0.005 ± 0.034 (-0.205–0.205)	0.003 ± 0.041 (-0.251–0.239)	P < 0.001
Ooplasm vs PVS area ratio	0.807 ± 0.052 (0.467–0.973)	0.816 ± 0.063 (0.399–0.982)	P < 0.001
Ooplasm vs ZP major axis ratio	0.702 ± 0.033 (0.514–0.846)	0.709 ± 0.039 (0.494–0.907)	P < 0.001
Ooplasm vs PVS area ratio–relative cohort	− 0.005 ± 0.048 (-0.327–0.302)	0.003 ± 0.059 (-0.46–0.294)	P < 0.001
ZP circularity	0.841 ± 0.063 (0.639–0.905)	0.847 ± 0.061 (0.57–906)	P < 0.001
Ooplasm vs ZP perimeter ratio	0.706 ± 0.028 (0.557–0.858)	0.711 ± 0.034 (0.515–0.874)	P < 0.001

### Performance of mask model

On a test set of 11,757 images, the Auto-sklearn model displayed an AUC of 0.63, a sensitivity of 0.38, and a specificity of 0.76. On the same test set, the LightGBM model displayed an AUC of 0.63, a sensitivity of 0.51, and a specificity of 0.66. While the two models demonstrated similar performance in terms of AUC and were both superior at differentiating the negative class, the sensitivity and specificity of the LightGBM classifier is more balanced. The Auto-sklearn model is also an ensemble, whereas the LightGBM is a single model, making it simpler to interpret and work with. Thus, it was selected as the final model for the classification task, and subsequent Principal Component Analysis (PCA) and SHAP (SHapley Additive exPlanations) analyses are based on the LightGBM model.

PCA reduced the number of included features from 47 to 9, capturing 95% of the explained variance. With the reduction to nine features, the LightGBM model displayed a significantly different and slightly lower performance with an AUC of 0.62, a sensitivity of 0.66, and a sensitivity of 0.51 than the LightGBM with all 47 features (p < 0.001, DeLong test) on the same test set (11,757). Therefore, reducing the data dimensionality did not seem to improve model performance, and may have resulted in the loss of important features relevant to the classification task.

**Figure 2:**
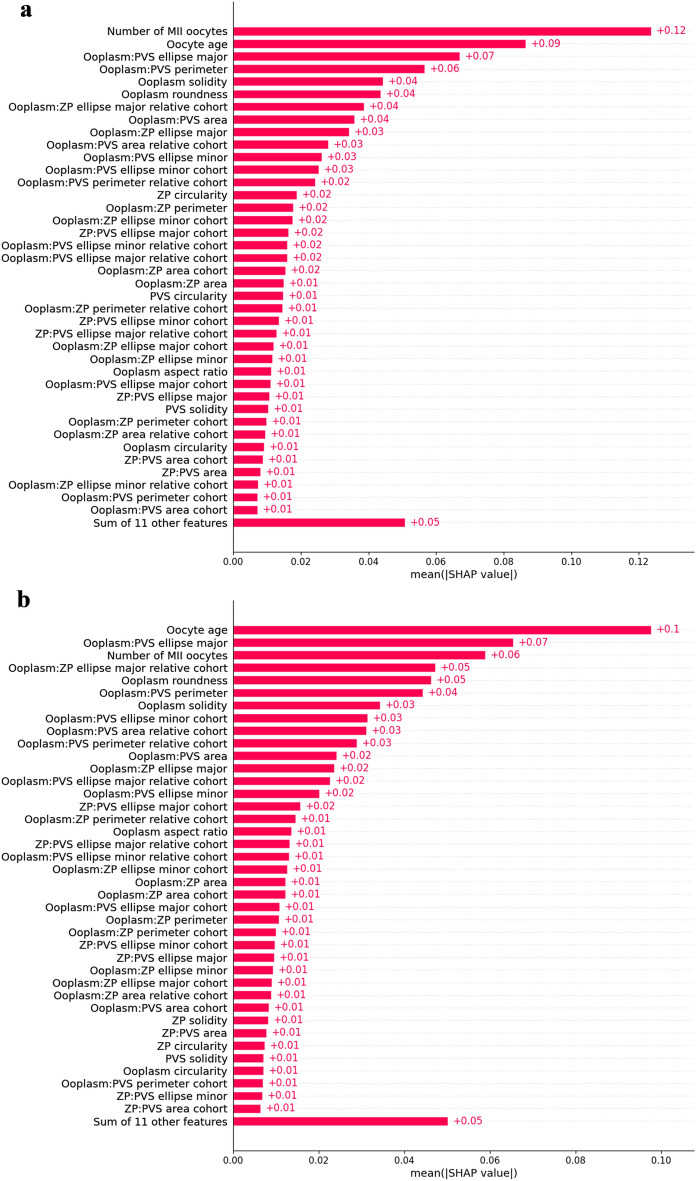
Mean Shapley values, ranking features used by the LightGBM mask model by importance to model predictions. (**A**) Mean Shapley values across entire model development dataset. (**B**) Mean Shapley values across external validation dataset.

Mean absolute Shapley values (MASV) across the entire dataset are summarized in Fig. 2. The clinical features of age and number of mature oocytes are shown to be the most important to global model performance, but other measurable features specific to individual oocytes such as the major axis ratio and perimeter ratio between the ooplasm and PVS, ooplasm solidity, and ooplasm roundness are also relevant.

**Figure 3:**
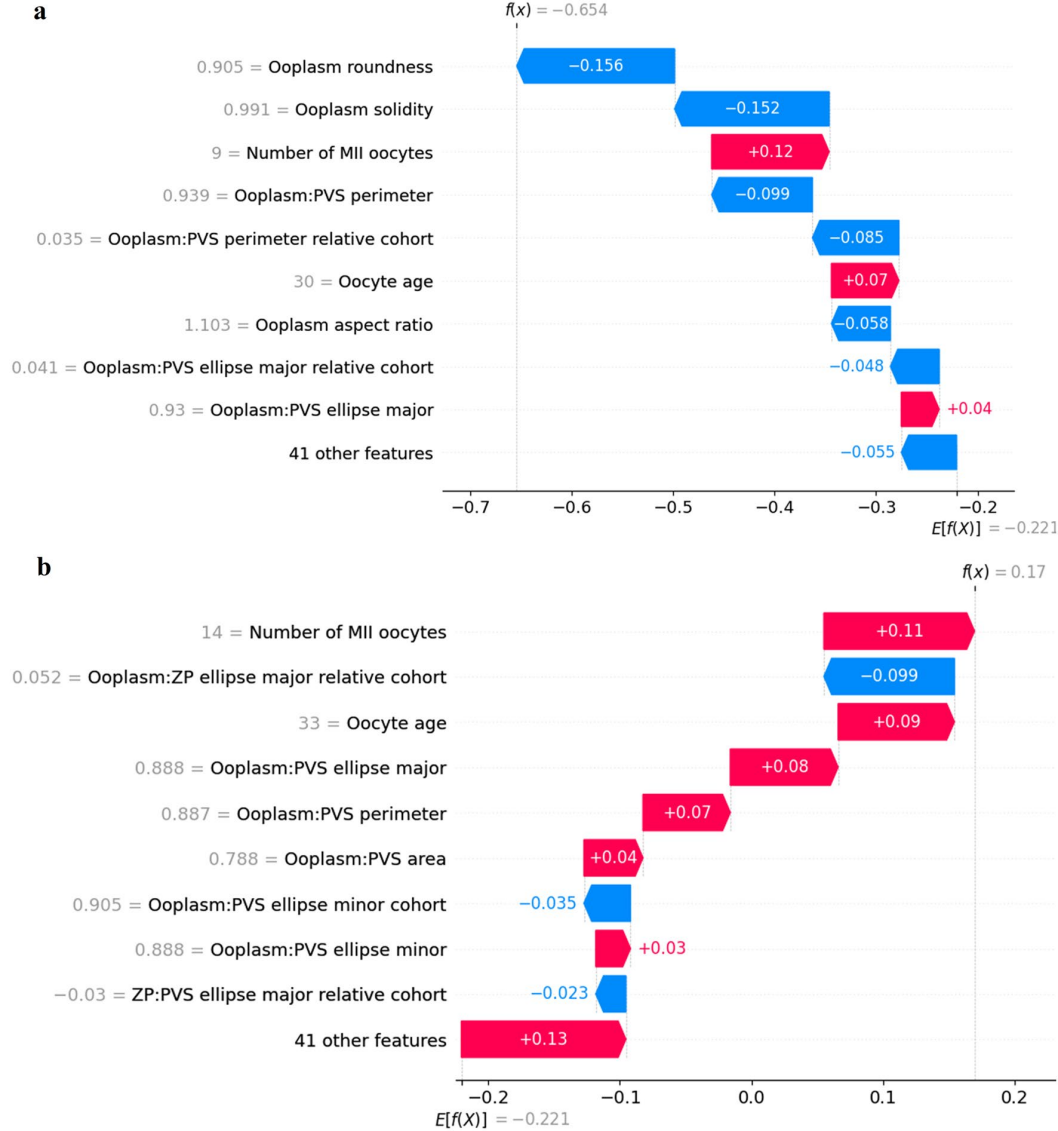
Waterfall plots visually demonstrating how model predictions are made. (**A**) Negative prediction. Starting from a prior expectation of − 0.221, the Shapley values of the features used by the model are added up to generate a value f(x) =  − 0.654—the logit of the model output that is then inputted into the sigmoid function to generate a prediction probability. (**B**) Positive prediction. Starting from a prior expectation of − 0.221, the Shapley values of the features are added up to generate the logit of the model output, f(x) = 0.17.

Additionally, SHAP values can be used to explain individual predictions. The waterfall plots in Fig. 3 provides examples of explanations for a negative prediction and a positive prediction, and visual demonstrations of how the SHAP values of each feature shifts the model output from the prior expectation (the average value of the model output of all the data) to the final model prediction. The prior expectation for the model is − 0.221, and adding up all the SHAP values results in a value f(x), which is the logit of the model output. To obtain the prediction probability, the f(x) is inputted into the sigmoid function. Thus, when the logit is zero, the prediction probability is 0.5 (the threshold); when the logit is positive, the prediction is positive; and when the logit is negative, the prediction is negative.

Removing all ooplasm-associated features as inputs resulted in the most drastic reduction in AUC on the test set, from 0.63 to 0.57, which was determined as statistically significant by the paired DeLong test (p < 0.001). Removing all extracytoplasmic-associated features resulted in small but significant changes of AUC on the test set: 0.62 for PVS (p < 0.01) and 0.64 for ZP (p < 0.01). Thus, the ooplasm appears to hold the most information relevant to the model’s discriminative ability, while inclusion of the ZP does not appear to aid model predictions. Removing clinical features (i.e. oocyte age and number of oocytes in the cohort) results in a small but significant reduction in AUC to 0.62 (p < 0.001). Removing cohort features (i.e. cohort averages and relative cohort features) increases the AUC on the test set to 0.64 but not significantly (p = 0.09).

### Performance of ensemble model

On the same set of 11,757 images used to test the LightGBM classifier model, the ensemble model (consisting of the LightGBM and DL ConvFormer model) achieved an AUC of 0.67, a sensitivity of 0.52, and a specificity of 0.7. For the ConvFormer model alone, AUC dropped to 0.66 and specificity to 0.65, while sensitivity increased to 0.56. Consistent with the ConvFormer architecture, combining the strengths of both CNN models and transformer models, the ConvFormer model outperforms our previously described CNN model^11^. The difference in AUC between the ensemble and ConvFormer model, while modest, was statistically significant as determined by the paired DeLong test (p < 0.001)—indicating adequate performance of the DL models, but with important contributions from the LightGBM model.

### Subgroup analysis

**Figure 4:**
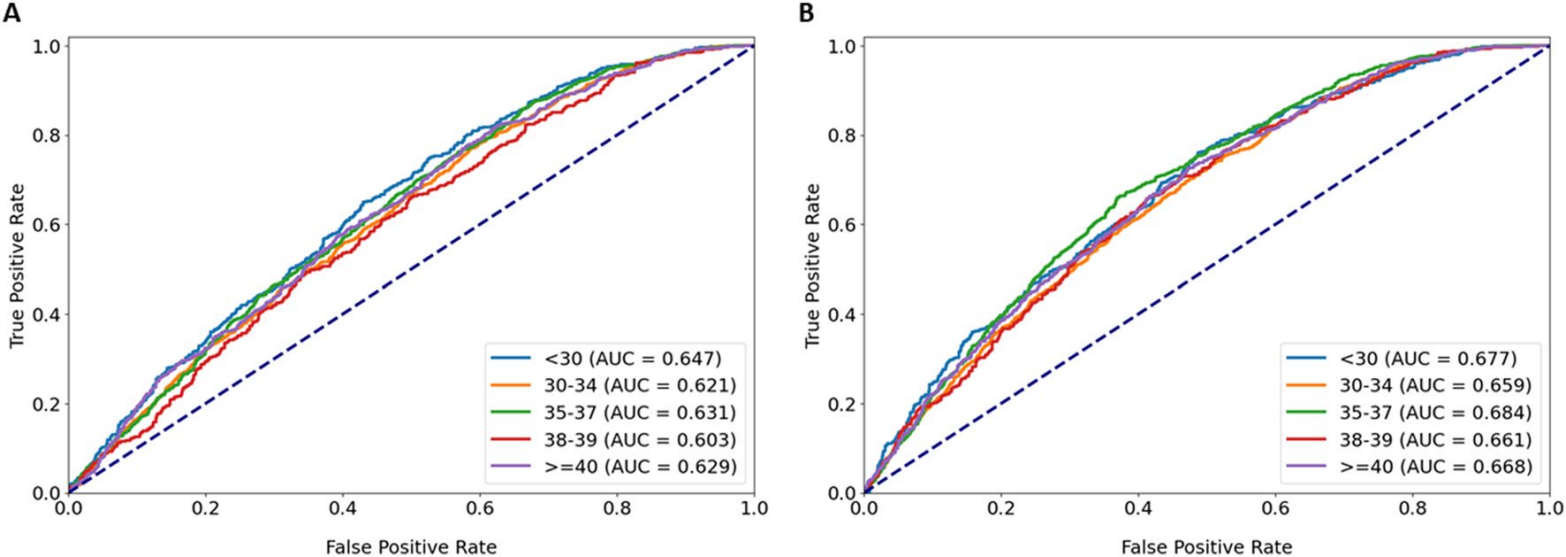
Subgroup analysis by patient age group for the mask and ensemble model. (**A**) Subgroup analysis by age group for the mask model displayed significant difference in performance only for the 38–39 age group (AUC 0.6) compared to model performance on the entire dataset (AUC 0.63) (p < 0.05, DeLong test). (**B**) Subgroup analysis by age group for the ensemble model displayed no significant differences in performance between the age groups and the entire dataset.

Subgroup analysis by age group for the mask model (visualized in Fig. 4a) revealed that only the 38–39 age group displayed a performance (AUC 0.6) that significantly differed from baseline performance (AUC 0.63) (p < 0.05, DeLong test). However, while the AUC for the age group ≥ 40 did not deviate from baseline, the sensitivity and specificity were 0.1 and 0.94 respectively, indicating poor performance on correctly predicting the positive class. This may be due to the mask model using age as a feature, which would make the negative class easier to predict for the model. For the ensemble model, no significant deviations from baseline in performance were observed (visualized in Fig. 4b). The sensitivity and specificity for the ≥ 40 age group were also less imbalanced (0.3 and 0.84 respectively)—thus, image analysis may contribute additional information that facilitates predicting the positive class. More in-depth information on the subgroup analysis for age group are available in Supplementary Tables S2 (mask model) and S3 (ensemble model).

**Figure 5:**
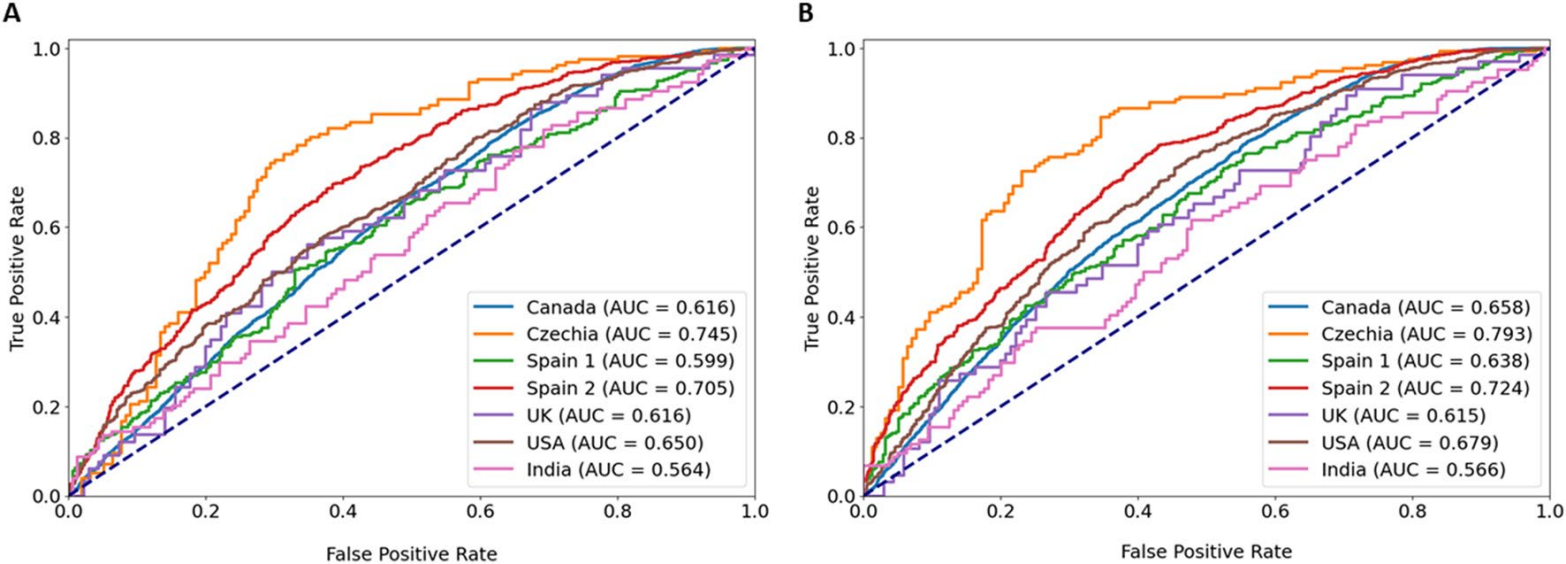
Subgroup analysis by clinic location for the mask and ensemble model. (**A**) Subgroup analysis by clinic location for the mask model displayed significantly higher performance for the Spain 2 clinic (AUC 0.71, p < 0.01 DeLong test) and the Czechia clinic (AUC 0.75, p < 0.01 DeLong test) compared to performance on the entire dataset (AUC 0.63). Performance on the Canada clinic displayed significant difference (AUC 0.62, p = 0.0496 DeLong test), however, with a borderline effect. B) Subgroup analysis by clinic location for the ensemble model displayed significant differences in performance for the Spain 2 (AUC 0.72, p < 0.01 DeLong test), Czechia (AUC 0.79, p < 0.01 DeLong test), and India (AUC 0.57, p < 0.01 DeLong test) clinic locations compared to the entire dataset (AUC 0.67).

Subgroup analysis by clinic for the mask model (Fig. 5a) displayed significantly higher performance on Spain 2 (AUC 0.71, p < 0.01 DeLong test) and Czechia clinic (AUC 0.75, p < 0.01 DeLong test) in comparison to the performance on the entire test dataset (AUC 0.63). Additionally, performance on the Canadian clinic location displayed statistical significance (AUC 0.62, p = 0.0496 DeLong test) compared to performance on the entire dataset. However, this p-value suggests a borderline effect. For the ensemble model (AUC 0.67), significant differences were displayed between the Spain 2 clinic (AUC 0.72, p < 0.01 DeLong test) and Czechia clinic (AUC 0.79, p < 0.01) location, as well as the Indian clinic location (AUC 0.57, p < 0.01 DeLong test) (Fig. 5b). More in-depth information on the subgroup analysis by clinic are available in Supplementary Tables S4 (mask model) and S5 (ensemble model).

### External validation

The mask model achieved an AUC of 0.63, a sensitivity of 0.56, and a specificity of 0.63. The ConvFormer model achieved the same AUC, but a more imbalanced sensitivity and specificity of 0.71 and 0.50 respectively. The ensemble model displayed a significantly higher AUC (0.65) than both the mask and ConvFormer models (p < 0.001 for both), and a sensitivity of 0.65 and a specificity of 0.57. Importantly, the AUC of the mask model remained stable on a new dataset, while performance of the ConvFormer and ensemble models dropped. Comparison of the MASV plots for the whole dataset and the external dataset reveal that seven of the top ten most important features (70%) overlap (i.e. oocyte age; number of MII oocytes; major ellipse ratio, perimeter ratio, and area ratio relative to the cohort between the ooplasm and PVS; major ellipse between ratio between the ooplasm and ZP; and ooplasm roundness and solidity).

## Discussion

In the present work, the LightGBM prediction model (mask model), utilizing extracted features and patient factors as inputs, was able to classify oocytes by competence to develop into a blastocyst with an AUC of 0.63, a sensitivity of 0.51, and a specificity of 0.66. Many image analysis models in the medical field aim to automate the work of medical specialists who are already highly proficient at identifying lesions, with often life-threatening consequences for the patient as a result of incorrect predictions, thus necessitating high performance metrics to be clinically relevant. It is significantly more difficult for human experts to predict downstream reproductive outcomes from evaluating gametes or embryos, due to the presence of many confounding variables that are difficult to quantify or control for (e.g., maternal factors following transfer of the embryo)—thus, even an AUC between 0.6 and 0.8, which may lack relevance for other medical fields, could be considered clinically useful in the fertility space. Several AI studies evaluating embryos for prediction of outcomes such as aneuploidy, clinical pregnancy, and live birth have achieved an AUC within this range^16–22^.

In the present study, AI was utilized to automate the process of measuring known objective morphometric descriptors of other cells for the oocyte^12^. Evaluation of other biological specimen in the IVF lab (e.g. sperm and embryos) typically involves visual inspection, but this approach has not been successful for oocytes^8^. Reasons for such difficulty in qualitatively assessing the oocyte may be attributed to the inherent subjectivity in the task, the rarity of particular morphological abnormalities (known as dysmorphisms), and the possible oversimplification of their biological significance. For example, a given dysmorphism may have a different origin in one oocyte over another or may be better tolerated due to genetic or environmental factors. Additionally, the oocyte is far less dynamic than the motile sperm or dividing, multicellular embryo, which complicates the discovery of non-invasive markers of quality. A quantitative assessment of the oocyte has several advantages, including greater objectivity, greater generalizability (every oocyte can be measured, but not every oocyte will possess dysmorphisms), and improved interpretability (as cellular shape can reflect cellular function or pathology).

There are few studies applying AI to oocyte evaluation. One group proposed an algorithm called MOMA (MII oocyte morphology analysis) that measured ZP thickness, PVS width, and ooplasm area in input images of human oocytes following segmentation and compared these measurements to baseline values obtained from morphologically normal oocytes^23^. MOMA was utilized to assess deviations from baseline measurements of oocytes considered “morphologically normal” rather than correlate any features to clinical outcomes. However, it is difficult to understand the clinical relevance of deviations from baseline, or of the baseline itself.

A later study by the same group aimed to refine oocyte segmentation through the development of two possible segmentation algorithms: one involving binary segmentation, in which pixels were mapped onto either the region of interest (ooplasm, ZP, or PVS) or background, and the second involving multiclass segmentation, in which the image was segmented into four classes (ooplasm, ZP, PVS, or background)^24^. Consistent with our findings, multiclass segmentation was shown to outperform binary segmentation by better handling class imbalances (where the PVS was most challenging to segment due to its area being much smaller than the background). Accuracy of oocyte segmentation is critical to downstream analyses. As measurements of different oocyte regions occur at a very small scale, this leaves a lower tolerance for errors in measurement. Notably, our results suggest that there may be a smaller tolerated range of values for the morphometric parameters investigated for oocytes that successfully developed into blastocysts, while oocytes that failed to develop into blastocysts showed a wider range.

Another group described a pipeline where images of oocytes were first segmented into five classes (background, ooplasm, ZP, polar body, and residual cumulus cells), from which 24 features were extracted, and finally, feature vectors were used to classify oocytes as viable or nonviable (defined as the ability of the oocyte to become a well-developed embryo)^25^. This was the only study involving automatic segmentation of the oocyte that assessed the link between extracted features and clinical outcomes, with the most significant feature being the number of polar bodies present. However, the clinical value of this finding is greatly limited by the small dataset used, consisting of only 103 oocytes after exclusion of poor-quality images, and the ability to consistently detect and segment the polar body.

To our knowledge, no prior study has investigated morphometric parameters of the meiotically mature oocyte with relation to clinical outcome in such detail. However, previous work does provide evidence that size and shape of the oocyte is important. Sufficient size would likely be relevant to the oocyte’s functionalities in storing substrates that support early embryonic development, and consistent with this reasoning, some studies have demonstrated that parameters such as oocyte diameter and volume are linked to embryo quality^26,27^. More elongated oocytes, termed “ovoid oocytes”, are associated with abnormal cleavage patterns during early embryo development^28^. While most of the features investigated displayed significant differences between oocytes belonging to the blastocyst-positive and blastocyst-negative groups, particular features were determined to be of greater importance by the mask model. Although age is a known predictor of oocyte quality at a population level due to differences in chromosomal integrity, it is not useful to the younger patient population, nor can it provide personalized insights to the older patient population^29–31^. The importance of age is demonstrated by the mask model, as removing it as an input feature results in reduced model performance. However, it lacks relevance in ranking oocytes within a cohort, as age is the same for all oocytes and is utilized in conjunction with other features relevant to both individual oocytes and oocyte cohorts.

The importance of the ooplasm to model predictions is expected as the ooplasm and its enclosing plasma membrane play an important role by transitioning into the zygote, then dividing to form the cleavage-stage and eventually blastocyst-stage embryo. Extra-cytoplasmic defects (i.e. of the PVS and ZP) may have a comparatively modest impact on oocyte quality. Indeed, previous studies have demonstrated that PVS width and ZP thickness correlate to reproductive outcomes^32–34^. However, in our experiments, the removal of the ZP mask did not negatively impact performance of the classifier model. The process of ICSI (which was performed in all oocytes included in the study) bypasses the required interactions that occur in natural conception or conventional IVF between the ZP and the sperm, therefore, it is likely not as critical to blastocyst development in this application. Curiously, despite being the highest ranked features in predicting blastocyst development, the removal of clinical features resulted in only a small (though still significant) reduction in performance. A possible reason for this is that there is a smaller loss in information compared to the removal of ooplasm-related features, which resulted in a more drastic reduction in performance.

Further, the proposed workflow is an example of “interpretable” AI, which is experiencing growing demand, especially in critical domains such as medicine. Model interpretability is a complex issue lacking a unifying framework across domains and assessing it in the context of oocyte evaluation is complicated by the absence of a standard of care. Here, we define interpretability as the model synthesizing and performing computations with higher accuracy in a comprehensive manner. The model’s ability may exceed that of a human’s due to the high volume of the data involved. Further work is necessary to improve interpretability of the model, by elucidating how specific features may influence developmental competence of oocytes, likely through studies performed on model organisms. For example, we observed that oocytes that develop into blastocysts tend to have a lower aspect ratio than those that do not, but presently we can only speculate how this may be biologically relevant for the oocyte based on past research on other cell types (e.g. could influence cytoskeletal organization or mechanosensing)^12^.

Additional challenges regarding extrapolating from other cell types is that research is often conducted on cells that interact with other cells within the context of a tissue or organ, emphasizing the importance of oocyte-specific studies to be applicable to IVF-ICSI cycles.

Interpretability becomes especially relevant when comparing traditional ML models, which rely on predefined features, to DL models, which learn from the raw data. The mask model described here achieved a lower AUC (0.63) than the ConvFormer DL model (0.66) or our previously described DL model (AUC 0.64)^11^. The trade-off for interpretability may be lower performance of a model, which could be considered acceptable depending on the application of the model or the preferences of different users (for example, scientific research may favour interpretability to gain potentially actionable insights that can inform areas of further study). The improved performance of the ensemble model further highlights the limitations of the mask model, which uses size and shape in addition to clinical parameters, but not textural information. By analyzing images without prior feature engineering, the DL model is capturing additional information related to oocyte quality that is imperceptible to the human eye. The further improvement of the ensemble model (consisting of both the mask and DL model) (AUC 0.67) indicates that the combination of measurable and (currently) non-measurable features contribute to assessing oocyte quality and are thus important considerations in oocyte assessment models. Additionally, a DL model may not be able to extract all the information relevant to oocyte quality (e.g., size; shape), and the mask model therefore helps the DL model by focusing on these additional aspects. The workflow described here can potentially be implemented both in scientific research and in clinical practice. However, external validation of the mask, DL, and ensemble models on a new dataset demonstrated that the mask model was able to maintain its performance, while the DL and ensemble models displayed reduced performance. This observation may suggest that incorporating measurable features could make models more generalizable.

Our study has several limitations. First, the model is only applicable to ICSI cycles, as images of denuded oocytes are only available for ICSI cycles. For conventional IVF cycles, oocytes are typically not denuded until post-fertilization, at the zygote stage, thus it was not practical to collect data from these cycles to develop the model. Furthermore, due to the retrospective nature of most of the data collection, there were incomplete details for microscopes and imaging systems, making it difficult to understand how possible differences in image acquisition affected model performance. However, the wide variety of imaging equipment captured in the data also makes the model applicable across a variety of lab settings. An intriguing application of the discussed workflow could be to elucidate the effects of cryopreservation on oocyte quality. As it has been previously demonstrated that oocytes undergo morphometric changes post-thaw, with certain changes (i.e. enlarged whole oocyte diameter; PVS shrinkage) resulting in reduced reproductive potential, models could be trained and tested on a new dataset of oocytes pre- and post-thaw following the workflow described here to investigate these effects more comprehensively^35^.

## Conclusions

In this study, a workflow involving image segmentation and classification to address the clinical gap of non-invasive, interpretable oocyte evaluation was proposed. An FCBFormer multiclass segmentation model paired with a LightGBM model to classify oocytes showed the best results—achieving an AUC of 0.63 and a more balanced sensitivity and specificity. This approach also augmented the performance of an ensemble model additionally incorporating a DL model, from an AUC of 0.66 to an AUC of 0.67, likely reflecting an ability to combine measurable and unmeasurable oocyte features to predict blastocyst development. External validation indicated the mask model was more robust than the DL or ensemble models on new datasets.

## Materials and methods

**Figure 6:**
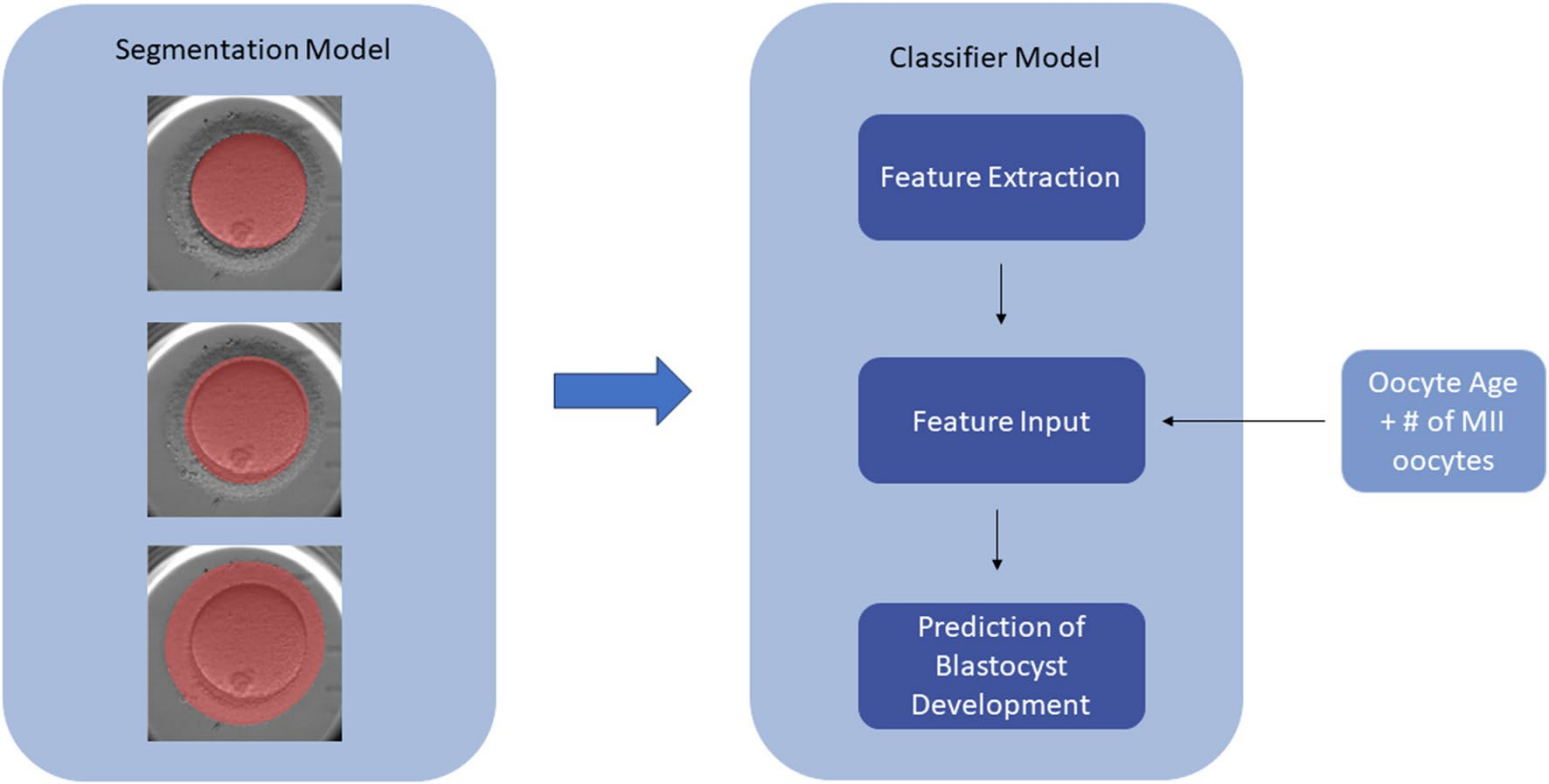
The proposed workflow utilizes two models—the first creates masks for each image of the oocyte, which is then used along with clinical variables as inputs into a classifier model (mask model) to generate a prediction of blastocyst development.

Herein, the development of two separate but related models to evaluate static images of denuded MII oocytes is described. The first model performs multiclass segmentation of the oocyte into ooplasm, ZP, and PVS, and the resulting masks are utilized as inputs for the second model—a classifier model (referred to as mask model) that extracts features from the masks and generates predictions of whether an oocyte develops into a blastocyst (Fig. 6). We summarize how the performance of the mask model is assessed and methods of determining the significance of the features it uses to make predictions. Finally, the mask model is compared to an ensemble model incorporating an additional DL model.

### Ethical approval

Research methods were all performed in accordance with relevant guidelines and regulations, and in accordance with the Declaration of Helsinki. Ethics and IRB approvals were obtained for the clinics involved from Veritas (2022-2547-13359-1), FutureLife Scientific Advisory Board, and Indira IVF Hospital Institutional Ethics Committee (IIHPL-UDR/RCT/004_2022), as necessary. Under the guidelines of the Human Fertilization and Embryology Authority (HFEA), studies on oocytes in the UK do not require a research license. Each participating centre reviewed the study protocol and provided approval. All subjects contributing their oocyte images to the study provided written informed consent to data sharing for research purposes prior to undergoing ICSI. Datasets were anonymized to protect patient privacy before being shared.

### Development of segmentation model

Images of 7792 denuded MII oocytes were utilized for the development of our segmentation model. 3258 images were obtained from five globally dispersed fertility clinics (located in Canada, UK, Spain, Czechia, and India). An additional 3138 images were obtained from an open-source dataset licensed under a Creative Commons Attribution 4.0 International^36^. The open-source data contained oocytes from various species—sea urchin, mouse and humans, however, only images of human oocytes, consisting of a combination of metaphase I (MI) and metaphase II (MII) maturation stages, were utilized. The resulting dataset included microscope and time-lapse (EmbryoScope and Geri) images of MI and MII human oocytes immediately pre- and post- ICSI. The task of manually assigning ground truth labels (ooplasm, PVS, and ZP) for 4654 images using the Computer Vision Annotation Tool (CVAT) was divided between three senior embryologists. This step was not necessary for the open-source dataset, which already possessed annotations for the ooplasm. Annotations of ZP and PVS for the open-source images were automatically generated by Oocytor, a Fiji Plugin tool capable of segmenting oocyte contours from multiple species, and then manually reviewed^36^. Of the 3138 open-source images, 380 samples with incorrect labels were removed during error analysis, leaving 2758 to be used. A final combined dataset of 7412 images of denuded oocytes underwent a split of approximately 60:20:20 to generate the model training, validation, and test sets—consisting of 4453, 1476, and 1483 images respectively.

A model with Fully Convolutional Branch TransFormer (FCBFormer) architecture was developed with PyTorch to perform multi-class segmentation of the oocyte^37,38^. We initially built a model of U-Net architecture, first to segment each region of the oocyte individually (background vs. region of interest), then in a second iteration to segment all four classes (background, ooplasm, PVS, ZP). The shift from binary segmentation to multi-class segmentation was justified by poorer performance on the PVS, a finding consistent with another study^24^. U-Net models are commonly applied to the task of medical image segmentation, due to their ability to extract both high-level contextual information (e.g. size, shape) and finer details that allow them to generate accurate segmentations^39^. The incorporation of skip layers in the U-Net architecture allows for the propagation and therefore the preservation of information from earlier layers to later layers in the network, which facilitates effective learning even with smaller datasets. However, the convolutional operator in CNNs such as U-Nets acts locally, making it challenging for these models to capture long-range dependencies. In contrast, transformer models efficiently make use of long-range dependencies to learn global context^40^. Thus, the FCBFormer architecture was recently proposed to exploit the merits of both fully convolutional neural networks and transformer models.

The FCBFormer model described here consists of a fully convolutional branch and transformer branch. The architecture of the transformer branch is Pyramid Vision Transformer v2—pre-trained on ImageNet—which is effective at dense prediction tasks (predicting at pixel-level) through generating multiscale feature maps and has the additional advantage over other transformer models in its ability to represent local features^41–43^. Both the input image and the segmented image generated by the model are resized to 352 by 352, the dimensions the FCBFormer architecture was developed on^38^.

Several augmentations were implemented prior to training using Torchvision^44^. Colour jitter with a brightness factor and a contrast factor uniformly sampled from 0.8 to 1.2 with a 0.7 probability was applied to vary the brightness and contrast of samples, respectively. Random JPEG compression with quality uniformly sampled from 35 to 100%, with a 0.5 probability was also applied, to vary the amount of detail present in each sample. Image sharpness was randomly adjusted by a factor of 2 with a probability of 0.5. Images also underwent random posterization with a 0.5 probability by reducing two bits for each color channel. To improve image contrast, the histogram equalization technique was applied randomly with a probability of 0.5. The order of the augmentation steps was random, and images were normalized to an interval of − 1 to 1.

The model was trained on 4453 images for 40 epochs using a batch size of 24 and Lion optimizer with an initial learning rate of 0.0001^45^. The Lion optimizer is memory-efficient and outperforms other optimizers such as Adam. Focal Loss was selected as the loss function, as it addresses class imbalance in the training set and facilitates the model’s ability to efficiently learn challenging or under-represented classes–relevant in the case of the oocyte, as the PVS is smaller than the other regions^46^.

To assess the accuracy of the FCBFormer model’s segmentation on the test set, intersection over union (IoU) scores—which determine the percentage of overlap between the ground truth and the model’s predictions—were calculated for each image and averaged to give a mean score for the ooplasm, ZP, and PVS. IoU scores were calculated separately for each mask to permit the monitoring of performance on each region of the oocyte, using the following formula:

1 $$IoU\left( {Y_{label} ,Y_{pred} } \right) = \frac{TP}{{TP + FP + FN}},$$

where ${Y}_{label}$ is the ground truth, which was manually assigned or checked by embryologists, and ${Y}_{pred}$ is the predicted masks. $TP$ is the true positive, which represents the intersection between the ground truth and model prediction; $FP$ is the false positive which represents the area where the ground truth is negative, but the prediction is positive; and $FN$ is the false negatives, representing the area where the ground truth is positive, but the prediction is negative.

### Development of mask model

51,831 static images of denuded MII oocytes with known blastocyst development outcomes, collected from seven clinics across six geographical locations (Canada, USA, Spain, Czechia, India, and UK), were used to develop a binary classification model (herein, referred to as the mask model) utilizing extracted features in addition to patient characteristics (i.e. oocyte age and number of MII oocytes) as inputs, with the output being a prediction of blastocyst positive or blastocyst negative. 2058 images of this dataset were utilized to develop the segmentation model described above. Images were captured either immediately pre- or post-ICSI, from various microscope setups (e.g. Leica, Nikon, Olympus, and Zeiss microscopes; with a designated Basler acA3088-57 camera) and time-lapse (e.g. Embryoscope, Geri) systems. All images were two-dimensional, single-plane, taken within a magnification range of ×200– ×400. Greater details on the imaging systems were unavailable due to the retrospective nature of the data collection. 6793 patients of mean age 37.1 ± 4.3 undergoing 8089 ICSI cycles contributed to the dataset. Conventional IVF cycles were not included in the model, as oocytes are not denuded during conventional IVF prior to insemination. Oocyte age (mean 36.0 ± 4.5 years) was used as a clinical feature in place of patient age to account for donor oocytes. Oocyte cohorts consisted of a mean 6.9 ± 4.6 oocytes. The dataset underwent a split of approximately 60:20:20, with 29,262, 10,812, and 11,757 images allocated between the training, validation, and test sets respectively. The dataset underwent splitting at the patient level rather than at the oocyte level to prevent data leakage. 20,927 oocytes had a ground-truth outcome of blastocyst positive (40.4% of the dataset), while 30,904 were labelled as blastocyst negative (59.6%). A blastocyst was defined as a day 5–7 (post-fertilization) embryo with a minimum Gardner grade of 1CC. Low-quality blastocysts were not filtered out. Dataset details are found in Supplementary Table S6.

The oocyte features to investigate were selected in accordance with morphometric descriptors commonly measured in two-dimensional cell shape analysis^12^. An ellipse was fitted to each mask to calculate the major ($${L}_{maj})$$ and minor ($${L}_{min})$$ axis, while perimeter $$\left({L}_{p}\right)$$, area $$\left(A\right)$$, and area of the convex hull, $${A}_{hull}$$ , were calculated from the masks themselves–these measurements were obtained using inbuilt OpenCV functions. These features were used in downstream calculations to define the aspect ratio, roundness, circularity, and solidity of each oocyte region, as summarized in Table 1. Ratios between two different masks (e.g. ooplasm vs ZP) for major axis, minor axis, perimeter, and area–here, termed relative features—were calculated and averaged for the entire cohort of oocytes. For each oocyte, cohort relative features were calculated by subtracting the cohort average from the value of the relative feature—thus, negative values for a given cohort relative feature indicate that the value of the relative feature for the oocyte under consideration is lower than the average of the cohort. Area of the convex hull was only used to calculate solidity and was not compared between oocyte regions. Altogether, 47 features were extracted to use as inputs for the mask model, encompassing relative features, average cohort features, cohort relative features, and mask-specific features. Relative features (e.g. ooplasm vs ZP area ratio) were used as inputs over absolute features (e.g. ooplasm area), due to their greater generalizability, which allowed the model to achieve a more balanced performance between clinics. Extracted features were also statistically compared between the group of blastocyst-positive and blastocyst-negative oocytes using the Welch’s *t*-test.

We experimented with two approaches to addressing the task of oocyte classification. The first approach was to use the Python-based automated machine learning toolkit, Auto-sklearn, which leverages Bayesian optimization, removes the need for the user to select algorithms and perform hyperparameter tuning, and constructs ensembles^47,48^. The Auto-sklearn model was constructed from an ensemble of extra trees classifiers—a type of machine learning model consisting of decision trees, where each decision tree randomly learns from the training set—and Gradient Boosting classifiers—where a strong model is created by combining several weak models, with each weak model correcting the mistakes of previous models. The second approach was training and evaluating a model with the light gradient boosting machine (LightGBM) framework. The LightGBM framework applies the technique of gradient boosting to quickly and efficiently train a collection of decision trees and is designed to be capable of handling large datasets and using less memory. To evaluate these two approaches, the performance metrics of area under the curve (AUC), sensitivity, and specificity were calculated.

To address the high dimensionality and potential interdependencies of the 47 features included in the mask model, a PCA was conducted prior to model training for comparison to a model trained without PCA. PCA attempts to reduce data dimensionality while maintaining the most relevant information, which can potentially improve the resulting model performance. Model performances were compared by AUC with a paired DeLong test^49^.

Feature importance with respect to blastocyst formation was assessed using the SHAP method. MASV across all samples in the dataset were used as a measure of the input features’ importance relative to one another, where a higher value is indicative of greater importance to the model’s prediction. SHAP values also facilitate the understanding of how each feature impacts a specific prediction. We additionally assessed the impact of removing the following features on model AUC: features related to each mask, clinical features, and cohort features. Changes in AUC were assessed for significance with the paired DeLong test^49^.

### Ensemble model

To assess if the problem of oocyte evaluation benefits from incorporating DL or if a single ML model is sufficient, an ensemble model consisting of the described LightGBM model and a DL model of ConvFormer architecture was constructed^50^. The ConvFormer architecture combines the strength of convolutional neural networks in capturing local details and the strength of transformer models in learning long-range dependencies to effectively classify images. It is pre-trained on ImageNet^41^.

The ConvFormer model was trained and validated on 48,363 and 10,812 denuded MII oocyte images, respectively, with associated outcomes from seven clinics. This dataset comprises of the same images utilized for the mask model development described above. Images underwent data augmentation using the AugMix method, which eliminate the need for hyperparameter tuning^51^. Pixels outside the ooplasm, PVS and ZP were set to zero to avoid the model paying attention to irrelevant parts of the image, such as residual cumulus cells or background noise. Images were cropped by an algorithm trained with Faster-RCNN and cropped images were resized to 224 by 224^52^. Binary cross entropy loss and an SGD optimizer with momentum of 0.9 and weight decay of 7.9e-5 were used to train the model, and the learning rate was adjusted using a cosine annealing schedule with warm restarts^53^. The model was trained for 40 epochs with a batch size of 128. To balance the positive and negative classes of the training dataset, samples of the minor class were oversampled to the same size of the major class for each clinic.

AUC, sensitivity, and specificity were all calculated for the ensemble model. Additionally, AUC of the DL model with and without the LightGBM was compared for statistically significant differences with the paired DeLong test.

### Subgroup analysis

Subgroup analyses by clinic and age group were performed to assess clinical relevance of the mask model and ensemble model for different patient populations (i.e. by geographic location and age). The age groups chosen for analysis were < 30 (n = 1607), 30–34 (n = 3515), 35–37 (n = 2646), 38–39 (n = 1594), and ≥ 40 (n = 2395) years, based on the age-related statistical decline of live birth in ICSI cycles^31,54^.

### External validation

A dataset of 9789 MII oocytes with known laboratory outcomes was obtained from a single Spanish clinic, independent of model development. 140 oocytes were removed from the analysis due to unknown blastocyst development outcomes (i.e., were only cultured until the cleavage stage prior to embryo freezing or transfer; in the case of transferred embryos, had unknown or negative implantation outcomes, as a positive implantation outcome would indicate in vivo blastocyst development). The impact of oocyte age on quality is especially pronounced after age 40, however this represents a significant percentage (17%) of the dataset. Therefore, we excluded oocytes > 43 years old, as the chances of live birth have been observed to fall below 5% in this age group^54^. The final dataset consisted of 9346 oocytes (mean age 31.0 ± 7.8 years), corresponding to 909 patients. 4691 of the oocytes developed into blastocysts (50.2%), while 4655 (49.8%) failed to develop into blastocysts. Performance of the mask model and ensemble model were evaluated on this dataset for AUC, sensitivity, and specificity, and AUC values were compared with the paired DeLong test.

### Supplementary Information

Supplementary Table S1.

Supplementary Table S2.

Supplementary Table S3.

Supplementary Table S4.

Supplementary Table S5.

Supplementary Table S6.

## Data Availability

The data collected from clinics for the present study is not publicly available due to ethical and data privacy considerations, and the authors lack authorization to release it. Data was anonymized before use. The open source data used in this study is available on Zenodo (https://zenodo.org/records/6502830)^36^.

## Code availability

The underlying code for the model described in this study is not publicly available for proprietary reasons.
