# Supplementary material for: Segmentation of mature human oocytes provides interpretable and improved blastocyst outcome predictions by a machine learning model
Source: Sci Rep. 2024 May 8;14:10569. doi: 10.1038/s41598-024-60901-1 (PMC11078996; doi:10.1038/s41598-024-60901-1)
Supplement: Supplementary file 1 — Supplementary Table S1. [file 41598_2024_60901_MOESM1_ESM.docx]

**Supplementary Table 1.** All morphometric measurements for oocytes that did and did not develop into blastocysts in model development

|  | **Feature** | **Mean ± sd (Range) for Blastocyst Positive Samples** | **Mean ± sd (Range) for Blastocyst Negative Samples** | **p-value** |
| --- | --- | --- | --- | --- |
| Ooplasm shape and contour descriptors | Ooplasm aspect ratio | 1.039 ± 0.037 (1.0 – 2.007) | 1.045 ± 0.045 (1.0 – 2.083) | P < 0.001 |
|  | Ooplasm circularity | 0.844 ± 0.065 (0.62 – 0.909) | 0.849 ± 0.062 (0.468 – 0.909) | P < 0.001 |
|  | Ooplasm roundness | 0.963 ± 0.031 (0.484 – 0.999) | 0.958 ± 0.037 (0.469 – 1.000) | P < 0.001 |
|  | Ooplasm solidity | 0.993 ± 0.002 (0.942 – 0.996) | 0.993 ± 0.003 (0.85 – 0.996) | P < 0.001 |
| PVS shape and contour descriptors | PVS aspect ratio | 1.053 ± 0.057 (1.000 – 2.139) | 1.054 ± 0.061 (1.0 – 2.446) | P < 0.01 |
|  | PVS circularity | 0.842 ± 0.066 (0.582 – 0.906) | 0.849 ± 0.063 (0.513 – 0.907) | P < 0.001 |
|  | PVS roundness | 0.951 ± 0.044 (0.454 – 0.999) | 0.950 ± 0.046 (0.406 – 1.0) | P < 0.01 |
|  | PVS solidity | 0.993 ± 0.001 (0.957 – 0.996) | 0.993 ± 0.002 (0.926 – 0.996) | P < 0.001 |
| ZP shape and contour descriptors | ZP aspect ratio | 1.054 ± 0.049 (1.0 – 1.851) | 1.055 ± 0.051 (1.0 – 1.994) | P < 0.01 |
|  | ZP circularity | 0.841 ± 0.063 (0.639 – 0.905) | 0.847 ± 0.061 (0.57 – 906) | P < 0.001 |
|  | ZP roundness | 0.95 ± 0.039 (0.524 – 0.999) | 0.949 ± 0.041 (0.498 – 0.999) | P < 0.01 |
|  | ZP solidity | 0.994 ± 0.002 (0.933 – 0.997) | 0.994 ± 0.002 (0.914 – 0.997) | P < 0.001 |
| Relative features | Ooplasm vs ZP area ratio | 0.501 ± 0.039 (0.311 – 0.804) | 0.509 ± 0.048 (0.268 – 0.809) | P < 0.001 |
|  | Ooplasm vs PVS area ratio | 0.807 ± 0.052 (0.467 – 0.973) | 0.816 ± 0.063 (0.399 – 0.982) | P < 0.001 |
|  | ZP vs PVS area ratio | 1.615 ± 0.088 (1.175 – 2.071) | 1.609 ± 0.094 (1.214 – 2.093) | P < 0.001 |
|  | Ooplasm vs ZP perimeter ratio | 0.706 ± 0.028 (0.557 – 0.858) | 0.711 ± 0.034 (0.515 – 0.874) | P < 0.001 |
|  | Ooplasm vs PVS perimeter ratio | 0.897 ± 0.031 (0.679 – 0.993) | 0.902 ± 0.037 (0.629 – 1.011) | P < 0.001 |
|  | ZP vs PVS perimeter ratio | 1.271 ± 0.035 (1.129 – 1.438) | 1.269 ± 0.037 (1.114 – 1.441) | P < 0.001 |
|  | Ooplasm vs ZP major axis ratio | 0.702 ± 0.033 (0.514 – 0.846) | 0.709 ± 0.039 (0.494 – 0.907) | P < 0.001 |
|  | Ooplasm vs PVS major axis ratio | 0.892 ± 0.039 (0.658 – 0.981) | 0.899 ± 0.045 (0.599 – 0.998) | P < 0.001 |
|  | ZP vs PVS major axis ratio | 1.271 ± 0.038 (1.05 – 1.437) | 1.269 ± 0.04 (1.089 – 1.459) | P < 0.001 |
|  | Ooplasm vs ZP minor axis ratio | 0.712 ± 0.029 (0.559 – 0.924) | 0.716 ± 0.035 (0.52 – 0.971) | P < 0.001 |
|  | Ooplasm vs PVS minor axis ratio | 0.904 ± 0.029 (0.703 – 0.998) | 0.907 ± 0.035 (0.637 – 0.997) | P < 0.001 |
|  | ZP vs PVS minor axis ratio | 1.27 ± 0.039 (1.069 – 1.503) | 1.268 ± 0.042 (1.027 – 1.524) | P < 0.001 |
| Cohort averages | Ooplasm vs ZP area ratio | 0.504 ± 0.031 (0.394 – 0.652) | 0.506 ± 0.032 (0.33 – 0.656) | P < 0.001 |
|  | Ooplasm vs PVS area ratio | 0.811 ± 0.038 (0.613 – 0.931) | 0.813 ± 0.039 (0.568 – 0.943) | P < 0.001 |
|  | ZP vs PVS area ratio | 1.612 ± 0.067 (1.404 – 1.899) | 1.612 ± 0.069 (1.355 – 1.937) | 0.315 |
|  | Ooplasm vs ZP perimeter ratio | 0.708 ± 0.022 (0.624 – 0.794) | 0.709 ± 0.022 (0.581 – 0.809) | P < 0.001 |
|  | Ooplasm vs PVS perimeter ratio | 0.899 ± 0.022 (0.772 – 0.964) | 0.9 ± 0.023 (0.739 – 0.984) | P < 0.001 |
|  | ZP vs PVS perimeter ratio | 1.269 ± 0.026 (1.181 – 1.369) | 1.269 ± 0.027 (1.162 – 1.376) | 0.479 |
|  | Ooplasm vs ZP major axis ratio | 0.706 ± 0.025 (0.604 – 0.804) | 0.707 ± 0.025 (0.543 – 0.87) | P < 0.001 |
|  | Ooplasm vs PVS major axis ratio | 0.896 ± 0.027 (0.741 – 0.968) | 0.897 ± 0.028 (0.655 – 0.998) | P < 0.001 |
|  | ZP vs PVS major axis ratio | 1.269 ± 0.028 (1.161 – 1.379) | 1.269 ± 0.029 (1.147 – 1.382) | 0.627 |
|  | Ooplasm vs ZP minor axis ratio | 0.714 ± 0.021 (0.629 – 0.821) | 0.715 ± 0.022 (0.533 – 0.839) | P < 0.001 |
|  | Ooplasm vs PVS minor axis ratio | 0.905 ± 0.019 (0.817 – 0.967) | 0.906 ± 0.02 (0.732 – 0.976) | P < 0.001 |
|  | ZP vs PVS minor axis ratio | 1.269 ± 0.027 (1.157 – 1.415) | 1.269 ± 0.028 (1.152 – 1.422) | 0.123 |
| Cohort relative features | Ooplasm vs ZP area ratio | -0.007 ± 0.054 (-0.288 – 0.573) | 0.005 ± 0.069 (-0.425 – 0.496) | P < 0.001 |
|  | Ooplasm vs PVS area ratio | -0.005 ± 0.048 (-0.327 – 0.302) | 0.003 ± 0.059 (-0.46 – 0.294) | P < 0.001 |
|  | ZP vs PVS area ratio | 0.002 ± 0.036 (-0.229 – 0.166) | -0.001 ± 0.039 (-0.234 – 0.257) | P < 0.001 |
|  | Ooplasm vs ZP perimeter ratio | -0.004 ± 0.028 (-0.168 – 0.209) | 0.002 ± 0.035 (-0.237 – 0.220) | P < 0.001 |
|  | Ooplasm vs PVS perimeter ratio | -0.003 ± 0.026 (-0.199 – 0.148) | 0.002 ± 0.032 (-0.258 – 0.148) | P < 0.001 |
|  | ZP vs PVS perimeter ratio | 0.001 ± 0.018 (-0.086 – 0.117) | -0.001 ± 0.019 (-0.107 – 0.125) | P < 0.001 |
|  | Ooplasm vs ZP major axis ratio | -0.005 ± 0.034 (-0.205 – 0.205) | 0.003 ± 0.041 (-0.251 – 0.239) | P < 0.001 |
|  | Ooplasm vs PVS major axis ratio | -0.004 ± 0.034 (-0.217 – 0.159) | 0.002 ± 0.039 (-0.323 – 0.175) | P < 0.001 |
|  | ZP vs PVS major axis ratio | 0.001 ± 0.02 (-0.139 – 0.135) | -0.001 ± 0.022 (-0.134 – 0.111) | P < 0.001 |
|  | Ooplasm vs ZP minor axis ratio | -0.002 ± 0.031 (-0.161 – 0.289) | 0.002 ± 0.036 (-0.236 – 0.247) | P < 0.001 |
|  | Ooplasm vs PVS minor axis ratio | -0.001 ± 0.026 (-0.184 – 0.126) | 0.001 ± 0.03 (-0.256 – 0.128) | P < 0.001 |
|  | ZP vs PVS minor axis ratio | 0.001 ± 0.023 (-0.134 – 0.124) | -0.001 ± 0.024 (-0.156 – 0.177) | P < 0.001 |
