# Supplementary material for: Segmentation of mature human oocytes provides interpretable and improved blastocyst outcome predictions by a machine learning model
Source: Sci Rep. 2024 May 8;14:10569. doi: 10.1038/s41598-024-60901-1 (PMC11078996; doi:10.1038/s41598-024-60901-1)
Supplement: Supplementary file 2 — Supplementary Table S2. [file 41598_2024_60901_MOESM2_ESM.docx]

**Supplementary Table 2.** Results of subgroup analysis by age group for the mask model.

| **Age** | **#Samples** | **#Blasts** | **Blast %** | **AUC** | **Sensitivity** | **Specificity** | **DeLong test p-value** |
| --- | --- | --- | --- | --- | --- | --- | --- |
| <30 | 1607 | 739 | 46.0% | 0.6469 | 0.6062 | 0.5956 | 0.3216 |
| 30-35 | 3515 | 1528 | 43.5% | 0.6205 | 0.6047 | 0.5506 | 0.2577 |
| 35-37 | 2646 | 1017 | 38.4% | 0.6311 | 0.6077 | 0.5678 | 0.9015 |
| 38-39 | 1594 | 653 | 41.0% | 0.6032 | 0.4809 | 0.6610 | **0.0499** |
| >=40 | 2395 | 819 | 34.2% | 0.6290 | 0.1026 | 0.9423 | 0.7766 |
| **DeLong test is comparing each group to the overall dataset* | | | | | | | |
