# Supplementary material for: Segmentation of mature human oocytes provides interpretable and improved blastocyst outcome predictions by a machine learning model
Source: Sci Rep. 2024 May 8;14:10569. doi: 10.1038/s41598-024-60901-1 (PMC11078996; doi:10.1038/s41598-024-60901-1)
Supplement: Supplementary file 3 — Supplementary Table S3. [file 41598_2024_60901_MOESM3_ESM.docx]

**Supplementary Table 3.** Results of subgroup analysis by age group for the ensemble model.

| **Age** | **#Samples** | **#Blasts** | **Blast %** | **AUC** | **Sensitivity** | **Specificity** | **DeLong test p-value** |
| --- | --- | --- | --- | --- | --- | --- | --- |
| <30 | 1607 | 739 | 46.0% | 0.6769 | 0.5751 | 0.6578 | 0.7911 |
| 30-35 | 3515 | 1528 | 43.5% | 0.6587 | 0.5569 | 0.6482 | 0.1598 |
| 35-37 | 2646 | 1017 | 38.4% | 0.6843 | 0.6077 | 0.6593 | 0.3305 |
| 38-39 | 1594 | 653 | 41.0% | 0.6607 | 0.5191 | 0.6908 | 0.3847 |
| >=40 | 2395 | 819 | 34.2% | 0.6684 | 0.3040 | 0.8432 | 0.6941 |
| **DeLong test is comparing each group to the overall dataset* | | | | | | | |
