# Supplementary material for: Segmentation of mature human oocytes provides interpretable and improved blastocyst outcome predictions by a machine learning model
Source: Sci Rep. 2024 May 8;14:10569. doi: 10.1038/s41598-024-60901-1 (PMC11078996; doi:10.1038/s41598-024-60901-1)
Supplement: Supplementary file 4 — Supplementary Table S4. [file 41598_2024_60901_MOESM4_ESM.docx]

**Supplementary Table 4.** Results of subgroup analysis by clinic for the mask model.

| **Clinic Location** | **#Samples** | **AUC** | **Sensitivity** | **Specificity** | **DeLong test p-value** |
| --- | --- | --- | --- | --- | --- |
| Canada  Czechia | 7298  312 | 0.6163  0.7453 | 0.5037  0.6154 | 0.6396  0.7564 | **0.0496**  **1.219e-4** |
| India | 263 | 0.5639 | 0.5481 | 0.5094 | 0.0605 |
| Spain 1  Spain 2 | 610  1619 | 0.5990  0.7050 | 0.3322  0.5403 | 0.7702  0.7270 | 0.1518  **1.505-7** |
| UK | 201 | 0.6163 | 0.3485 | 0.7826 | 0.6958 |
| USA | 1454 | 0.6500 | 0.5128 | 0.6755 | 0.2492 |
| **DeLong test is comparing each group to the overall dataset* | | | | | |
