# Supplementary material for: Segmentation of mature human oocytes provides interpretable and improved blastocyst outcome predictions by a machine learning model
Source: Sci Rep. 2024 May 8;14:10569. doi: 10.1038/s41598-024-60901-1 (PMC11078996; doi:10.1038/s41598-024-60901-1)
Supplement: Supplementary file 5 — Supplementary Table S5. [file 41598_2024_60901_MOESM5_ESM.docx]

**Supplementary Table 5.** Results of subgroup analysis by clinic for the ensemble model.

| **Clinic Location** | **#Samples** | **AUC** | **Sensitivity** | **Specificity** | **DeLong test p-value** |
| --- | --- | --- | --- | --- | --- |
| Canada  Czechia | 7298  312 | 0.6577  0.7933 | 0.4801  0.7308 | 0.7112  0.7500 | 0.0535  **5.788e-6** |
| India | 263 | 0.5656 | 0.4327 | 0.6164 | **0.0036** |
| Spain 1  Spain 2 | 610  1619 | 0.6384  0.7242 | 0.4252  0.5700 | 0.7605  0.7226 | 0.1269  **1.409e-4** |
| UK | 201 | 0.6146 | 0.4545 | 0.6889 | 0.1623 |
| USA | 1454 | 0.6793 | 0.6496 | 0.6037 | 0.6798 |
| **DeLong test is comparing each group to the overall dataset* | | | | | |
