# Supplementary material for: Segmentation of mature human oocytes provides interpretable and improved blastocyst outcome predictions by a machine learning model
Source: Sci Rep. 2024 May 8;14:10569. doi: 10.1038/s41598-024-60901-1 (PMC11078996; doi:10.1038/s41598-024-60901-1)
Supplement: Supplementary file 6 — Supplementary Table S6. [file 41598_2024_60901_MOESM6_ESM.docx]

**Supplementary Table S6.** Dataset description per clinic location.

| **Clinic Location** | **#Patients** | **#Cycles** | **Mean patient age ± sd** | **Oocyte age ± sd** | **Mean MII ± sd** | **Blast ratio** |
| --- | --- | --- | --- | --- | --- | --- |
| Canada | 4732 | 5689 | 36.6 **± 4.1** | 36.1 **± 4.1** | 7.0 **± 4.5** | 37.8% |
| Czechia | 105 | 105 | 36.1 **± 5.9** | 33.7 **± 5.7** | 7.0 **± 5.2** | 50.0% |
| India | 56 | 57 | 32.8 **± 5.0** | 31.9 **± 4.4** | 9.9 **± 6.5** | 38.6% |
| Spain 1 | 126 | 128 | 40.4 **± 4.1** | 32.5 **± 8.0** | 10.3 **± 3.6** | 49.4% |
| Spain 2 | 926 | 1175 | 39.1 **± 4.3** | 35.2 **± 5.5** | 5.3 **± 3.1** | 43.9% |
| UK | 40 | 40 | 34.3 **± 5.0** | 34.3 **± 5.0** | 7.0 **± 3.4** | 32.4% |
| USA | 792 | 905 | 37.4 **± 4.3** | 36.5 **± 4.3** | 7.5 **± 5.6** | 48.4% |
| **Total** | 6777 | 8099 | 37.1 **± 4.3** | 36.0 **± 4.5** | 6.9 **± 4.6** | **40.4%** |
